# The “Adipo-Cerebral” Dialogue in Childhood Obesity: Focus on Growth and Puberty. Physiopathological and Nutritional Aspects

**DOI:** 10.3390/nu13103434

**Published:** 2021-09-28

**Authors:** Carmine Bruno, Edoardo Vergani, Michele Giusti, Alessandro Oliva, Clelia Cipolla, Dario Pitocco, Antonio Mancini

**Affiliations:** 1 Dipartimento di Medicina e Chirurgia Traslazionale, Fondazione Policlinico Universitario A. Gemelli IRCCS, Univesità Cattolica del Sacro Cuore, 00168 Rome, Italy; carmine.bruno@outlook.it (C.B.); edoardo.vergani@outlook.it (E.V.); alessandro.oliva996@gmail.com (A.O.); dario.pitocco@unicatt.it (D.P.); 2 Servizio di Dietetica e Nutrizione Clinica, Azienda Sanitaria dell’Alto Adige, 39042 Bressanone, Italy; michele.giu93@gmail.com; 3 Dipartimento di Scienze della Salute della Donna, del Bambino e di Sanità Pubblica, Fondazione Policlinico Universitario A. Gemelli IRCCS, 00168 Rome, Italy; clelia.cipolla@policlinicogemelli.it; 4 UOSD Diabetologia, Fondazione Policlinico Universitario A. Gemelli IRCCS, 00168 Rome, Italy

**Keywords:** adipose tissue, adipokines, insulin-resistance, metabolic syndrome, growth, growth hormone deficiency, diet, personalized medicine

## Abstract

Overweight and obesity in children and adolescents are overwhelming problems in western countries. Adipocytes, far from being only fat deposits, are capable of endocrine functions, and the endocrine activity of adipose tissue, resumable in adipokines production, seems to be a key modulator of central nervous system function, suggesting the existence of an “adipo-cerebral axis.” This connection exerts a key role in children growth and puberty development, and it is exemplified by the leptin–kisspeptin interaction. The aim of this review was to describe recent advances in the knowledge of adipose tissue endocrine functions and their relations with nutrition and growth. The peculiarities of major adipokines are briefly summarized in the first paragraph; leptin and its interaction with kisspeptin are focused on in the second paragraph; the third paragraph deals with the regulation of the GH-IGF axis, with a special focus on the model represented by growth hormone deficiency (GHD); finally, old and new nutritional aspects are described in the last paragraph.

## 1. Introduction

Overweight and obesity in children and adolescents are overwhelming problems in western countries. Despite the concept of a tracking phenomenon, which predisposes such populations to develop or worsen obesity in adult age, various data suggest that complications can occur already in childhood [1], opening the problem of a definition of metabolic syndrome (MetS) in children. As described in detail in adults, MetS is a constellation of conditions, linked to increased cardiovascular risk, such as hypertriglyceridemia, hyperinsulinemia or type 2 diabetes mellitus (T2DM), hypertension, and nonalcoholic fatty liver disease (NAFLD) [2].

Overweight young people are likely to become overweight adults [3]. Body composition can be more relevant than body mass index (BMI) itself in determining the well-known complications; in Asia, indeed, T2DM can ensue at a lower degree of obesity in the young population [4]. However, in childhood, both fatty liver and increased intima thickness have been described [5,6]. Different anthropometric indexes have been associated with cardiometabolic risk in pre-school children [7]; similarly, some metabolic parameters, such as the percentile of serum uric acid, have been considered predictors of cardiometabolic abnormalities [8].

The criteria for a correct diagnosis of MetS in the pediatric population are still matters of debate [6].

The discussion of growth alteration in MetS can rely on two main topics: (1) the role of nutritional status in puberty induction; (2) the influence of low-grade inflammation (LGI) on the growth hormone (GH)-insulin-like growth factor (IGF)-1 axis. Both aspects are still incompletely understood, especially the second one, recently enriched with new information about inflammatory parameters, oxidative stress (OS), and adipokines and their reciprocal influences.

Adipocytes, far from being only fat deposits, are capable of endocrine functions, which assign them a leading role in neurohormones regulation involved in different processes [9]. The plasticity of adipocytes and their dialogue with the central nervous system (CNS) are attractive fields of investigation.

The concept of the “brain–adipose axis” was introduced by Shimizu [10] who proposed some criteria to identify molecules involved in this axis: the expression of endogenous molecules and/or their receptors both in hypothalamus and peripheral adipose tissue; their identification in plasma; a central activity of hunger/satiety regulation; a peripheral influence on adipocyte size and number. We prefer to use the term adipose-cerebral dialogue as the endocrine activity of adipose tissue seems to be a key modulator of CNS functions.

Even if the main target of adipokines is related to the control of appetite and energy metabolism, recent data, both in vitro and in experimental animals, suggest a wider spectrum of activities, including neurotrophic and neuroprotective ones. Excellent review articles have been published on this topic [11,12]. In humans, a fundamental field of investigations regards the role of adipokines in neurodegenerative diseases; a prominent example is Alzheimer’s dementia, which exhibits neuropathological features such as those of diabetes mellitus, and it is also defined as type 3 diabetes [13,14].

Focusing on the model of childhood obesity, we aimed to review the possible impact of nutrition, metabolic derangement, and adipokines on growth and puberty.

The peculiarities of major adipokines are briefly summarized in the first paragraph; leptin and its interaction with kisspeptin are focused on in the second paragraph; the third paragraph deals with the regulation of the GH–IGF axis, with a special focus on the model represented by growth hormone deficiency (GHD); finally, old and new nutritional aspects are described in the last paragraph.

## 2. Adipokines in Childhood Obesity

We previously described the role of different adipokines in childhood obesity [15], however, this field of investigation is rapidly growing. 

Leptin is by far the most important adipokine involved in the balance between nutritional status, adipose tissue function, and appetite regulation. It exerts central and peripheral effects. Its elevated levels in obesity are proportional to body weight increase, associated with a bizarre loss of effects on the hypothalamic control of appetite, configuring a condition described as leptin resistance. Leptin is also linked to insulin-resistance (IR), OS, and the development of NAFLD [16]. Table 1 summarizes the main features of adipokines and their relation to insulin-resistance; therefore, we further discuss some insight about childhood obesity and their relations with CNS.

### 2.1. Leptin 

Leptin involvement in metabolism is part of a complex network of signals determining the energy balance of the individual. It acts as a long-term regulator of the level of the body mass, particularly fat mass, thus representing a true indicator of the energy status of the individual. It has the delicate task of reporting energy status and metabolic behaviors toward structures that control the genetically and centrally determined set-point. A recent study in overweight children and adolescents showed significantly higher values of leptin in children with high BMI [40].

Reflecting the body fat reserves, leptin plays a crucial role in the regulation of numerous neuroendocrine functions, from energetic homeostasis to a variety of processes related to reproduction [41,42], bone function [43], cardiovascular system regulation [44], and immune function [45].

Once in circulation, bound to the transport protein Ob-Re (splice variant receptor Ob-R or LEP-R), leptin reaches its sites of action. Another subtype of leptin transporter ensures the crossing of the blood–brain barrier, in order to create a precise balance between blood concentrations of the hormone and the cerebrospinal fluid (CSF) levels, which are between 2 and 5% of serum values [46]. Multiple brain regions express LEP-R [47]; different isoforms have been detected by in situ hybridization studies: Ob-Ra and Ob-Rb are mainly hypothalamic, while Ob-Ra, Ob-Rc, and Ob-Rf predominate in the choroid plexus [48]. The hypothalamic ventromedial (HVM) and lateral (HL) nuclei present the highest concentration of LEP-R on various peptidergic neurons. The hormone acts through the action of neuropeptide Y (NPY) by depressing the activity of neurons recognized as the “neurons of the hunger”. In contrast, it stimulates nerve cells that synthesize peptides such as proopiomelanocortin (POMC) and the derived melanocortin, also known as α-melanocyte-stimulating hormone (α-MSH), and cocaine-and-amphetamine-regulated-transcript (CART), considered the “neurons of the satiety” [12,49]. Appetite regulation is sustained by a complex network where first-level neurons, in turn, influenced by nutritional stimuli (leptin), metabolic fuels, gastro-intestinal hormones (such as ghrelin and glucagon-like peptide 1), and neural signals mediated by the nervus vagus, project to second-order neurons that represent the effectors of hunger and satiety. The first order level, in summary, includes the orexygenic NPY neurons, also coexpressing the agouti-related peptide (AgRP) and the anorexigenic POMC/CART neurons [50]. α-MSH works via binding melanocortin 4 receptor (Mcr)-4. Extensive projections of α-MSH neurons in the arcuate nucleus reach the dorso-medial hypothalamic nucleus, the medial pre-optic area, and the anterior hypothalamus [51]. Some fibers also project to the paraventricular nucleus, the lateral hypothalamus, the posterior hypothalamus, and the central nucleus of the amygdala [50]. Another kind of Mcr, Mcr-3, has a less clear role; interestingly, it has been detected in the nucleus accumbens, which could be of interest in psychiatric disease [52] and opioid addiction [53]. Recently, important connections of POMC neurons have concerned somatostatin-producing Sst1.1 neurons in the pre-optical area. The latter exert a suppressive action on GH expression and release by pituitary somatotrope cells. Due to the expression of Mcr4 and the ability to respond to exogenous α-MSH, these Sst1.1 cells could be considered second-order neurons [54].

While these mechanisms represent the key leptin role as a homeostatic hormone in response to energy excess, other functions are related to hedonic feeding behavior; they involve interactions with endocannabinoid signaling, orexinergic neurons, and neurotransmitters such as dopamine and gamma-aminobutyric acid (GABA) [12,55,56,57].

The correlation between leptin and ghrelin should be noted. The two hormones are secreted independently of one another, but they act on the same target in the hypothalamus in an antagonistic way. Recent evidence shows that one of the major risk factors for the development of obesity is the inability of leptin to perform its metabolic functions of appetite suppression and promotion of energy expenditure. Both in humans and obese animals, however, higher blood levels of leptin have been found, suggesting a mechanism of reduced sensibility on targets, known as leptin resistance. Different pathophysiological mechanisms are capable of explaining this phenomenon. Among them, we recall the reduced transport of leptin across the blood–brain barrier, the intracellular signal attenuation, mutations of the genes for leptin or its receptor, intracellular OS, and inflammation [58]. We recently described progressive increasing levels from normal weight, overweight to obese children, also showing a correlation with plasma antioxidant capacity; we have suggested a possible role in the modulation of the response to OS in obesity [59]. In this regard, it is important to keep in mind that antioxidant defenses can be incompletely developed in children, with the scarce possibility to counteract oxidative damage [60]. 

Leptin has pleiotropic activities, influencing pituitary hormones, such as thyroid-stimulating hormone (TSH) and GH [49,61]. As far as GH is concerned, interesting data come from the model of leptin deficiency offered by ob/ob mice, which exhibit reduced linear growth and GH-IGF-1 axis activity, even with species-specific differences [62]. In this context, an indirect effect could be mediated by the above-cited leptin-POMC-Sst1.1 axis.

A correlation with thyroid function has also been described [59]; therefore, leptin seems to be a multifaced hormone, with further functions to be detected.

It is well known that lipolysis in white adipose tissue is guaranteed by a great number of adrenergic receptors expressed by the adipocyte [63]. Leptin can modulate the sympathetic effects on white adipose tissue; a pathway between CNS and adipose tissue, mediated by leptin, has been demonstrated to regulate lipolysis [64]. Moreover, some brain areas, including the arcuate nucleus, preoptic area, dorsomedial, and HVM, are greatly involved in brown adipose tissue thermogenesis [65]. Thermogenesis is related to the expression of uncoupling protein (UCP)-1: again, leptin has been demonstrated to be the mediator of UCP-1 expression and of the changes in the GH/IGF-1 axis induced by negative energy balance [66].

In our dissertation on the problem of obesity and growth, two main aspects of leptin physiology must be stressed:

- first, insulin drive on leptin synthesis and release, which can partly explain hyperleptinemia in obesity; experimental data in animals and in humans with a hyperinsulinemic–euglycemic clamp suggest that this phenomenon is observed with supraphysiological insulin levels in humans, with physiological levels in rodents [67].
- secondly, leptin-resistance, which develops in obese subjects, related to post-receptorial mechanisms [61], may be related to endoplasmic reticulum stress, hypothalamic inflammation, and autophagy [58].

**Figure 1:**
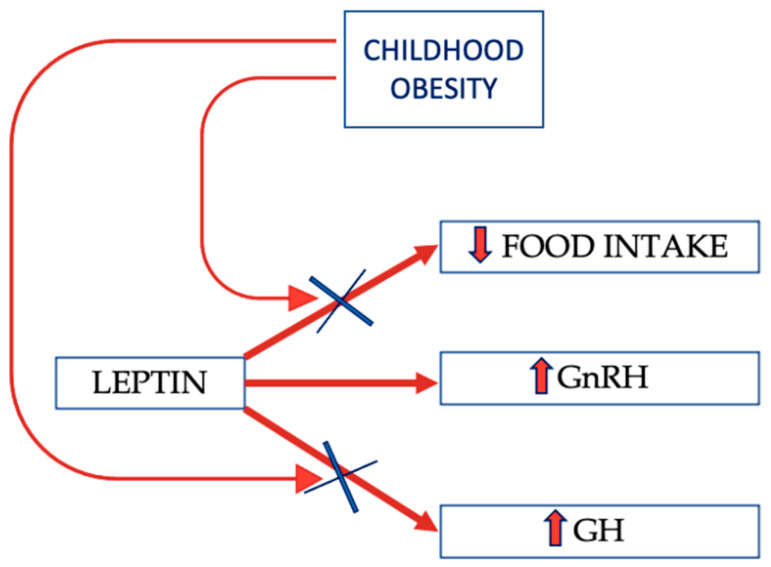
Physiological leptin effects on hypothalamic–pituitary axis; selective resistance can ensue in obesity, blocking some of them (see text). GH: growth hormone; GnRH: gonadotropin-releasing hormone.

An interesting model of acquired leptin-resistance is represented by excessive energy intake in larval zebrafish, in which a long-term caloric excess obtained by ad libitum/high-fat diet conditions induces obesity coupled with increased linear growth. In this model, the reduction in leptin-related effects induces a decrease in POMC neurons activation, in turn related to diminished somatostatin secretion and, consequently, GH increase and linear growth. This pathway, active in mammals, is, however, less pronounced than in fish as excessive caloric intake is related to fat storage and obesity development, so that other factors can overcome the above-cited pathway [54]. The relationship between POMC and GH seems to be regulated by a fine-tuning loop; in fact, in the mouse pituitary gland, IGF-1 is able to suppress GH synthesis and to stimulate POMC synthesis at the transcriptional level [68]. The author hypothesized an autocrine mechanism in somatotropic cells also linked to corticotrope function, but the connections between POMC and somatostatin could open new scenarios on the talk between GH system and hunger/satiety-related neurons. As discussed below, leptin-resistance seems to be tissue-selective, acting mostly on some specific leptin effects (Figure 1).

### 2.2. Resistin 

Also known as adipose tissue-specific secretory factor (ADSF) and found in inflammatory zone 3 (FIZZ3), it is expressed and secreted mostly by monocyte-macrophages in visceral adipose tissue [17].

Studies on its secretion in childhood obesity are still controversial; some studies have suggested a possible role under inflammatory conditions such as atherosclerosis [16,69,70,71].

Central effects of resistin include a modulation of orexigenic AgRP and NPY expression in response to fasting, and modulation of sympathetic nerve activity and pituitary GH secretion [72,73,74]. 

### 2.3. Chemerin

It is a chemoattractant protein, produced in the liver and adipose tissue, which interacts with a receptor (chemokine-like receptor 1, CMKLR1) located in adipocytes, endothelial cells, and inflammatory cells. Therefore, it is clearly linked to LGI and indexes of inflammations, as demonstrated in children [75]. It is involved in adipogenesis, both in adult and children obesity, and its levels are elevated [31,32], particularly when visceral adipose tissue is predominant; it is positively correlated with leptin and negatively with adiponectin [33]; children with low Vitamin D levels exhibited higher chemerin concentrations [76]. Chemerin has been recently shown to be a marker of NAFLD in children [77]. 

A central action in regulating appetite and in neuroprotection in experimental models has been described [78,79]. 

#### Vaspin

Vaspin is a serin-protease inhibitor belonging to the serpin family produced in the adipose tissue. It appears to reduce glucose tolerance, thus leading to IR. High levels of vaspin have been linked with obesity [80,81]. Chunyan reported a relation between vaspin and obesity. This adipokine is also interrelated with visfatin, and both have a regulatory effect on inflammation. In children with obesity, increased levels have been described; vaspin is directly associated with body weight, triglycerides levels, insulin concentrations, IR, and high systolic and diastolic blood pressure; on the contrary, it is negatively associated with adiponectin and endothelial function [82,83].

### 2.4. Adipocyte-Fatty-Acid-Binding Protein

Adipocyte-fatty-acid-binding protein (AFABP) is a fat-derived circulating protein; it plays a key role in the regulation of glucose and lipid homeostasis. AFABP is associated with IR, low high-density lipoprotein (HDL) cholesterol, and an elevation in C reactive protein (CRP) [82,84,85]. 

An in vivo study in mice demonstrated IR induction after AFABP administration [86].

AFABP is significantly higher in overweight/obese than lean children, and correlated with waist circumference and insulin independently from age, BMI, gender, and developmental stage; weight loss is effective in reducing AFABP levels [87].

### 2.5. Adiponectin

Adiponectin is a protein highly expressed in adipose tissue; its levels decrease in obese patients, particularly in those with abdominal obesity, while they seem to increase during weight loss. Recently, the link between low levels of adiponectin and MetS has also been shown in obese children and adolescents [88]. 

Adiponectin has been detected in the CSF with species-specific ratios with plasma [89]. The low molecular form (trimeric) can directly cross the brain–blood barrier [90] and reach its two receptors (AdipoR1 and AdipoR2) in the hypothalamus but also in the cortex, hippocampus, striatum, and thalamus [11]. Other than food intake modulation, it exerts a role in neurogenesis and neurotropism in rodents [4]. 

Interestingly, metabolic milieu influences adiponectin activities: the effects on POMC neurons are dependent on glucose blood concentration (activation at low glucose levels and inhibition at high glucose levels) [91]. On the contrary, adiponectin reinforces the GABAergic inhibition on NPY neurons with a glucose-independent mechanism [91]. Finally, a crosstalk with leptin has been hypothesized in dopaminergic neurons of the ventral-tegmental area [92]. 

Adiponectin also inhibits gonadotropin-releasing hormone (GnRH) neurons, with consequence on puberty [93]. It exerts in vitro cultured hypothalamic cells for both the inhibition of GnRH release and Kiss1 mRNA production [94,95]; taken together, this evidence underlines the link with negative energy balance and delayed puberty, as a specular model in comparison with obesity. 

### 2.6. Visfatin

It is not known if visfatin is produced directly by CNS cells or derived from circulation; however, it increases food intake, maintains nicotinamide adenine dinucleotide (NAD)+ homeostasis, and improves insulin sensitivity [11].

The role of visfatin in children and adolescents is still debated. A significant positive association between plasma visfatin and anthropometric parameters has been described by some authors [37,96,97], but this finding has not been confirmed by others [98,99,100].

An independent association between visfatin concentration and IR in healthy lean adults has been found [101], while previous studies have not confirmed these findings in physically inactive obese children and adolescents [98,99,100].

### 2.7. Apelin

While it is not conclusively demonstrated if apelin crosses the blood–brain barrier, the APJ receptor has been detected in the peripheral and CNS. Apelin involvement in learning and memory processes, in the neuroregulation of the hypothalamic–pituitary–adrenal axis, and in the neuroprotection against neuroinflammation by decreasing myeloperoxidase, IL-1, and TNFα, has been demonstrated through intraventricular administration in experimental models [11]. In a large Italian cohort study in children [102], apelin correlates negatively with age and pubertal status, but no relations have been demonstrated with body weight, unlike in adults; it has been hypothesized that lower apelin levels during puberty could be one of the factors involved in the physiological insulin-resistance of puberty [102]. 

## 3. Leptin–Kisspeptin Interplay in Pubertal Development

The “critical weight hypothesis” is based on the concept that a minimum body weight or fat percentage is necessary to start pubertal changes [103,104]. Nevertheless, in overweight/obese females, a trend toward earlier menarche has been detected, and estimated as 13 days in advance for each kilogram of body weight [105]. Gender-related differences have been described, with early signs of puberty in girls and a consequently short stature related to advanced skeletal maturation, whereas pubertal delay can be seen in boys [106]. Leptin levels increase before puberty in girls and their peak precedes the gonadotropins one. Overnutrition, inducing early hyperleptinemia, can be responsible for early puberty [107]. The ratio between leptin and adiponectin has been considered as a predictor of weight gain [108]. Leptin can also interact with adrenal function, as it is correlated with the androgen dehydroepiandrosterone (DHEA) [109].

In synergy with the above-described factors, other mechanisms, such as early estrogenization and the influence of hyperinsulinism, could be involved in puberal advance [106,110]. 

Interactions between leptin and kisspeptin in the determination of pubertal onset is well described in the literature, as leptin ultimately stimulates GnRH neurons via binding to its receptor on kisspeptin neurons.

Kisspeptin is a key regulator linking nutritional status and the GnRH–pituitary–gonadal axis. It is produced mainly by the arcuate nucleus in the hypothalamus and by other tissues, including ovaries. It exerts its effects on KISS1R/Kiss1r [111]. Its physiological activity is linked to other substances co-expressed in the same neurons of the antero-ventral periventricular nucleus (AVPV) in rodents and in the preoptic area in humans [112], with a contribution to the preovulatory gonadotropin surge only in the female sex; an augmenting effect of estradiol on this mechanism has been reported [113]. In rodents, estradiol and testosterone downregulate kiss1 mRNA in the arcuate nucleus, while only estradiol upregulates kiss1 mRNA in AVPV, which exhibits a greater number of these neurons, explaining the sexual dimorphism in pubertal timing also in humans [114,115,116].

Anyhow, kisspeptin is a regulator of GnRH pulsatility [117]. This regulation is modulated by some other peptides, such as neurokinin B and dynorphin. These two last neurosecretory hormones influence kisspeptin release in an opposite way [118]. Kisspeptin is an initiator of GnRH release even in the male population; its activity is exerted in a stronger and continuous modality, inducing a constant luteinizing hormone (LH) release and, consequently, a testosterone increase [117]. A sex-related dimorphism in kisspeptin levels has been demonstrated in obese adults [119] and children, with higher levels in females than male patients [59]. It has been shown that kisspeptin levels increase from puberty to adult age, while this pattern is not present in boys; moreover, prepubertal female obese children have been shown to have greater levels than nonobese ones, but no data have been reported on obese prepubertal males [119].

However, we have previously studied a cohort of prepubertal obese subjects; as a result, our data showed a sex dimorphism, suggesting that the difference is not related to the milieu of circulating steroids, but, on the contrary, could be related to a differential hypothalamic control already present before puberty [59]. 

Such a difference could develop in very early stages of life: several studies have indicated a sex dimorphism in the hypothalamic Kiss1-receptor in different animal species, including mice [120,121], rats [115], and Guinea pigs [122]. Furthermore, sexual differences were evidenced only in our obese group and not in normal weight controls. This result could be explained by the higher levels of leptin present in overweight and obese children, due to the stimulating effect of leptin on kisspeptin: variations in circulating leptin or the nutritional status influence kisspeptin expression [116,123,124], suggesting that kisspeptin has a key role in the link between nutritional signals and reproduction. Finally, we suggested that in prepubertal obese children, increased leptin could trigger an amplification of sex-related kisspeptin pattern [59].

The nutritional status and body fat percentage represent environmental factors in continuous interaction with the genetic background, thus explaining an important individual variability. Recently, via genome-wide association studies, new genetic loci have been shown to be involved in the timing of pubertal maturation, such as melanocortin-4 receptor, mitochondrial carrier 2, and mitogen-activated protein kinase 13 [118]. The role of adipokines is, anyhow, uncontroversial. 

Other than leptin, a negative regulator of kisspeptin is adiponectin, in turn negatively regulated by inflammatory cytokines, including tumor necrosis factor (TNF) α and interleukin (IL)-6 [125].

It must be reminded that, while the kiss1-receptor is expressed in adipose tissue, the pancreas, and gonads [126,127], kisspeptin is also expressed in different tissues, including the liver, pancreas, gonads, placenta [128,129], and human female adipose tissue [130]. The model of Kiss1-receptor knockout mice, which is characterized by obesity, reduced metabolism, and energy expenditure, reinforces the concept of pleiotropic actions of kisspeptin other than reproductive ones [129].

Finally, it seems presumable that reproduction development and function rely on a complex interplay between central (cited above) and periphery mechanisms, as several adipokines, such as omentin, resistin, visfatin, and chemerin, are also expressed in the ovaries [131,132,133], while resistin has been found in the rat testis [134], suggesting an interaction between central and peripheral machineries. 

## 4. GH/IGF-1 Axis: The Model of GH Deficiency

It is widely accepted that GH secretion, both in the basal condition and after stimulation tests, is blunted in obesity [135,136]. Low levels are related to a reduced amount of GH due to a flattening pulsatory secretion, rather than a low bursts frequency, and accelerated metabolic clearance rate. Low GH levels are associated with an alteration of body composition and cardiovascular risk parameters. Among these, a pro-atherogenic lipidic pattern in both sexes, higher levels of CRP, increased thickness of carotid intima-media, and low adiponectin levels have been described [137,138,139].

The reciprocal influence between functional GH deficiency and obesity are bidirectional. This is not surprising considering the role of the GH-IGF-1 axis on adipose tissue [140]: GH and IGF-1 receptors are present in all cytotypes of such tissue: preadipocytes and mature adipocytes, but also immune cells, fibroblasts, and endothelial cells [141,142,143]. GH influences all stages of adipocyte life, from differentiation to ageing; GH and IGF-1 favor lipolysis and counteract lipogenesis. Finally, GH has been hypothesized to induce “browning” of adipocytes, i.e., the proportion and thermogenic activity of beige adipocytes [144].

It is known that obese children exhibit reduced GH secretion while IGF-1 levels can be similar to normal-weighted ones [145]. Among the factors implicated in GH suppression, hyperinsulinemia is one of the most important [146].

IGF-1 is usually decreased in MetS and T2DM, due to the lack of an insulin inhibitory effect on hepatic production of IGF-binding protein-1. Moreover, IGF-1 secretion is reduced due to the IR as well [147]. It has been shown that GH, IGF-1, and insulin interact in the regulation of nutritional state and adipogenesis. GH has a direct effect on mature adipocytes, favoring lipolysis and the increased release and oxidation of free fatty acid [148]. Adult mice with partial IGF-1 deficiency presented a reduced expression of genes implicated in lipid metabolism and cholesterol synthesis. Low IGF-1 levels are associated with reduced insulin sensitivity [149], glucose intolerance, and T2DM [150,151]. Inflammatory cytokines may decrease IGF-1 circulating levels in experimental models [152]. Moreover, inflammatory cytokines hindered IGF-1 signaling via the phosphorylation of serin residues of insulin-related substrate (IRS) [153]. Consequently, beneficial effects of IGF-1 are blocked.

The receptor of ghrelin (growth hormone secretagogue receptor) is expressed in kisspeptin neurons in rats; although leptin stimulates kisspeptin, ghrelin suppresses kisspeptin expression in the arcuate nucleus [154]. Ghrelin levels in obese children are low while its receptor antagonist LEAP-2 is increased, favoring a further hyposecretory GH condition [155].

As a whole, these data hint that childhood obesity is associated with a reduction in GH-mediated effects. For this reason, it is possible to compare the “secondary” GH deficit seen in obesity to a primary one. Taking GHD as a model allows us to investigate the effects of GH on adipokines and adipose tissue.

In GH-deficient children, leptin levels have been reported to be unchanged [156,157,158] or higher [159] in comparison with controls. After a 12 month GH treatment, different results have been shown: the Ciresi group and Lopez group found lower leptin levels, while Meazza found increased ones.

Adiponectin levels in GHD were measured equal [156,159], higher [157,158], and even lower [160] than those in the controls before treatment, even though they remained unchanged after a 12 month treatment [156,157,158]. Resistin levels appeared higher [156,158], equal [157], or lower [158] in GHD when compared to controls. After treatment, resistin levels increased [148,161], slightly increased [156], decreased only at 6 months [157], or decreased at 12 months [158]. Finally, Ciresi et al. reported lower visfatin and unvaried omentin, retinol binding protein-4, and α-FABP in GHD children before treatment. After treatment, visfatin increased, omentin decreased, while RBP-4 and α-FABP remained unchanged.

The inhomogeneity between results hints that more factors, yet to be studied, have to be considered when evaluating GH effects on adipokines. Stawerska et al. proposed that IGF-1 bioavailability, in turn, related to the interaction between IGF-1 and IGF binding protein (IGFBP)-3, and not the bare GH, may be considered to better comprehend this phenomenon [159]. IGFBPs are a group of proteins that bind and modulate IGF activities. Six types, from IGFBP-1 through IGFBP-6, have been described in humans with several activities linked to IGFs binding and eventually IGFs-independent [162]. IGFBP-3 is the most prevalent in adult serum with a concentration five times greater than the others [163]. About 75–80% of serum IGFs form a ternary complex with IGFBP-3 (less often IGFBP-5) and an acid labile subunit. Other than enhancing the IGF-1 serum half-life, IGFBPs prevent the potential cross-reaction between IGF-1 and insulin receptors, a crucial event as IGF concentrations are high enough in the serum to cause hypoglycemic effects even given their lower affinity for the insulin receptors [164]. IGFBP-3 is mainly produced in the liver and its secretion is GH-dependent [164], thus indirectly related to nutritional status. IGFBP-1 is also synthesized in the liver and its expression is regulated by catabolic factors and hormones: starvation, hypoxia, stress, and glucocorticoids are promoting factors [165,166], while insulin is an inhibiting factor [167,168]. The functional role of IGFBP-1 under the condition of starvation is to reduce the rate of development and growth by binding to IGFs and inhibiting IGF activity [165,166].

**Figure 2:**
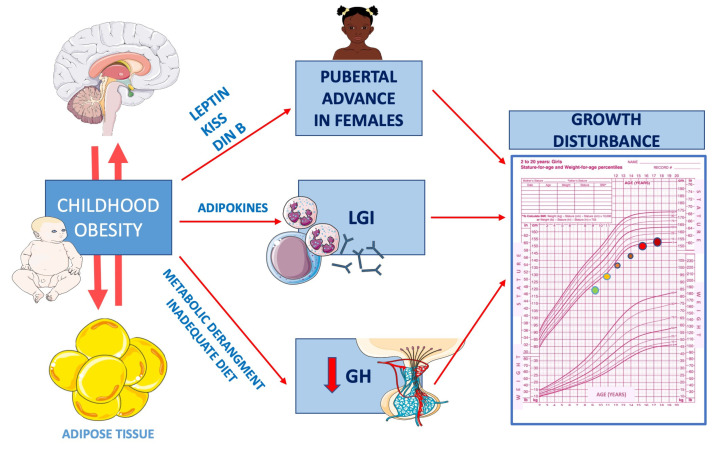
Mechanisms related to impaired growth in obese children. As explained in the text, childhood obesity influences growth and puberty acting via different mechanisms. Precocious activation of the leptin–kisspeptin axis is responsible for early-onset puberty, especially in female adolescents. Obese children present growth hormone hyposecretion. Systemic low-grade inflammation is also involved. GH: growth hormone; LGI: low-grade inflammation; KISS: kisspeptin; DIN B: dynorphin B.

Dividing GHD children by IGF-1 bioavailability, they found that children with low IGF-1/IGFBP-3 ratio had higher adiponectin and equal leptin and resistin than children with high IGF-1 bioavailability did. Moreover, from a clinical point of view, children with lower IGF-I/IGFBP-3 had a better metabolic profile [135,159] (Figure 2). 

## 5. Nutritional Aspects

As discussed above, overweight and obesity in children are among the most serious public health issues of this century, as they have reached epidemic proportions during recent decades. It is well known that children eat small daily amounts of fruit and vegetables while preferring snacks and soft drinks containing sugar and highly caloric and industrialized food. Moreover, new generations practice less sport and spend plenty of time performing sedentary activities [169]. The mechanisms underlying a positive energy balance (due to excessive calorie intake and low energy expenditure) are complex, including several of the pathways described above. Central and peripheral actions of adipokines, usually under a refined control, are disrupted in obese children [10]. The traditional approach of the problem deals with dietary consequences on BMI and anthropometric measures, while a new approach based on studies that explore the impact on hormonal response appears to be more intriguing, yet, nowadays, still inconclusive.

### 5.1. Traditional Approach

The cornerstone of treatment for childhood obesity is the modification of dietary and exercise habits [170].

#### 5.1.1. Dietary Approach: The Role of Carbohydrate and Fat Intake

Although there is not enough evidence that carbohydrates increase the odds of global obesity, carbohydrates adjustment is often the major component of weight-loss plans in childhood obesity [169,171,172]. From a physiological perspective, a low-carbohydrate diet (LCD), i.e., <40% of total energy, reduces caloric intake and body weight due to increased demands on other substrates (such as amino acids and free fatty acids) that, through gluconeogenesis or lipolysis, produce usable energy. The use of substrates other than glucose is more pronounced in low-carbohydrate and low-fat diets (LCFDs), where the waste of protein intake used for energy expenditure is not known. An interesting topic is represented by the evaluation of the effect on insulin-sensitivity, in turn related to adiponectin-insulin interplay, after LCD or LCFD in puberty, a period where an augmented insulin secretion (almost a doubling) is requested to counteract the physiologic decrease in insulin-sensitivity.

Growing evidence indicates that obesity outbreaks increased dramatically all over the world in correlation to the increased intake of refined carbohydrates and sugars in the era of industrial food. The World Health Organization (WHO) strongly recommends, in 2015 guidelines [173], the reduced intake of free sugars, for less than 10% of the total energy intake both in adults and children. Free sugars consist of all monosaccharides and disaccharides added to foods, plus sugar naturally present in honey, syrups, and fruit juices. The rich consumption of fructose, commonly underestimated, leads to pro-inflammatory signaling by the release in various adipokines, correlated to IR and consequently to an overexpansion of adipose tissue [174]. Chen et al. reported that excessive energy intake from fructose may elicit mitochondrial OS and decrease Thioredoxin 2 abundance, which is a vital factor for the viability of cells [175], and alters GLUT5, a major co-transporter of glucose, thereby promoting IR [176]. Sugar-sweetened drinks, such as carbonated soda, energy drinks, and highly sweetened coffees and teas, are frequently taken by adolescents, favoring fat deposition. Those products, in the last few years, were replaced on the market by sugar-free products. De Ruiter et al. showed that a masked replacement of sugar with sugar-free products significantly reduced weight and body fat gain in children [177]. Independently from total energy intake, a low-calorie sweeteners consumption is associated with increased obesity among adolescents [178]. 

Fat intake has to be considered too. Indeed, high fat intake is directly correlated with hepatic IR due to the activation of protein kinases that impair glucose uptakes in adipocytes and muscles [176]. 

However, Gibson et al. showed how little we know about the best dietary approach in childhood obesity. LCD, in children, are more effective than other protocols in the short term; nevertheless, no significant data on long-term effects are available [179].

Ketogenic diets (cKD) are increasingly being adopted for weight loss. During a 12 month follow-up of patients under cKD, De Amicis et al. [180] did not observe any change in leptin and ghrelin plasmatic levels independently from age and clinical conditions; thus, it seems presumable that cKD action does not rely on these peptide pathways. The actual clinical research on cKD is not enough to promote this regimen in all guidelines worldwide as a healthy strategy to weight loss in children.

#### 5.1.2. The Role of Lifestyle: Physical Exercise

Besides diets, lifestyle advice such as physical exercise and regular and various breakfast administrations are, especially in childhood, fundamental. The duty to change children’s habits should not only concern the family, but also policies and strategies in schools with projects and increased sessions for physical activity, food advertisings, specific food taxes, and more. Luley et al. [181] evaluated three additional strategies to reduce weight in obese families: an additional diet preferring carbohydrates having a low glycemic index, financial incentive, and telemonitoring of weight and physical activity. All the strategies worked on parents, while, surprisingly, children improved in terms of BMI independently from the strategy adopted, but strictly influenced by the collective family endeavor.

Diet modification alone is not a sufficient approach. When caloric intake decreases, metabolism slows down, resulting in a decreased calorie utilization and difficulty achieving the prefixed target. Therefore, exercise is an essential element for childhood obesity treatment [172].

Interestingly, when comparing different intervention methods in the management of obesity, the most effective approach was based on physical activity rather than dieting alone: the protocols relying on exercise seems to be more effective than those that employed just a hypocaloric diet [182]. A recent meta-analysis on a vast population of patients affected by obesity (*n* = 4815) showed that, despite a larger effect of diet on total body weight loss, exercise tends to have superior effects in reducing visceral adipose tissue, the “metabolically dangerous” one, as its secretomics is more inflammatory than the subcutaneous one [183,184]. As total body weight loss does not necessarily reflect changes in visceral adipose tissue, it is possible to conclude that physical exercise, particularly endurance and aerobic training, is an essential and unreplaceable element in lifestyle-interventions, when combined with diet.

### 5.2. New Approaches

The complex scenario of weight gain and loss has been recently enriched by new insights on LGI, OS, hormonal networks, adipokines, and gut microbiota.

#### 5.2.1. Hormones and Inflammation: A Viable Option?

The potential pro-inflammatory role of diet has been recently investigated: the “Children’s Dietary Inflammatory Index” (C-DII) has been shown to be correlated with adiposity and index of LGI (IL-6 and CRP and lower adiponectin/leptin ratio) at the age of 11 years [185].

Other than the general recommendations described, some dietary aspects are directly related to the hormonal mechanisms discussed in this review, especially leptin, GH, and LGI. As previously discussed, Aguirre et al. reported that the inhibition of IGF-1 causes severe risks to develop IR and obesity [147].

IGF-1 and insulin are strongly linked to each other, as observed during postprandial periods, where an increase in free circulating IGF-1 via the insulin-induced suppression of IGFBP-1 secretion and via dietary protein intake has been demonstrate d [186].

Ikeda et al. demonstrated that levels of hepatic IGF-1 remained high for 4 or 8 h after a protein-only diet intake with an influence on the circadian clock [186]. The same group suggested that the consumption of a high-protein breakfast could prevent obesity through the up-regulation of IGF-1, particularly in T2DM.

An interesting systematic review on the hormonal response to dietary treatment underlined that knowledge on this topic is still scarce. Some studies explored the modification of leptin, ghrelin, and adiponectin. In some cases, a dissociation between BMI changes and hormonal/metabolic picture has been described. While the correlation between leptin plasmatic levels and BMI is clear, evidence on adiponectin changes according to BMI is still inadequate. A possible bias source is represented by different adiponectin isoforms, as high-molecular-weight adiponectin has been regarded as the most representative form in children. A similar situation, but with a lower quality of evidence and controversial findings, can be seen for ghrelin, for which different isoforms have been recognized and the relation to the GH-IGF-1 axis has been poorly investigated [187]. Despite the burden of new findings on adipokines and their role on obesity and IR, a complete knowledge of the field is too far away to be considered realized. It is well known that exercise also plays a pivotal role. However, it has been shown that in obese children and patients with T2DM, acute exercise can induce a worsening in inflammation and OS; indeed, IL-6 levels, elevated under basal conditions in comparison to controls, further increased after exercise in T2DM, while myeloperoxidase, which was basally increased in T2DM, showed an exercise-induced increment in obese children. Other parameters showed a differential pattern, suggesting a dysregulation in response to physical activity [188]. Beneficial effects, on the contrary, are related to chronic training (see above) [189]. An interesting study in 132 healthy children showed a significant difference in OS markers between a group with low or high fitness with higher levels of total glutathione and oxidized glutathione and a lower reduced glutathione ratio in the first one [190]; beneficial effects have also been reported on parameters of bone mass, size, and structure [191]. It is presumable that the same concepts can be applied to the obese population. 

#### 5.2.2. The Paradigm Shift: Gut Microbiome and Obesity

The discovery and typing of gut microbiota have allowed in recent years a paradigm shift in world nutrition. Gut microbial alterations may cause metabolic consequences, such as additional energy sources and energy regulation, glucose-stimulated insulin secretion, and the release of peptide hormones that control appetite, contributing to obesity [180]. 

Unhealthy diets or food choice can promote an inflammatory condition strictly related to microbiome modifications. Some interesting new nutritional theories [192] are based on the individual microbiome structure, with the precise purpose of limiting specific groups of diet depending on microbiome composition, to establish and avoid the onset of obesity. 

A high-fat diet can change the upper small intestinal microbiome [193], and the transplantation of a healthy gut microbiome to the upper small intestine of individuals with metabolic syndrome increases insulin sensitivity [194]. In this regard, it is conceivable that the gut microbiome affects tissue insulin sensitivity. A recent proposed mechanism to explain the phenomenon relies on the production of taurochenodeoxycholic acid (TCDCA), which exerts, just as a hormone, its effects on farnesoid X receptor (FXR). The inhibition of FXR in the small intestine increases insulin sensitivity and glucose tolerance in rats [195,196]. Surprisingly enough, a high-fat diet in mice decreases Lactobacillus Gasseri species in the gut microbiome, the same species that expresses a bile salt hydrolase capable of transforming the dangerous TCDCA in chenodeoxycholic acid, a bile acid with unknown effects on insulin activities [197].

Furthermore, it is known that branched-chain amino acids (BCAAs) serum levels are higher in obesity and related to IR and T2DM [198,199]. Indeed, BCAAs restriction improves insulin sensitivity and muscle glucose disposal in Zucker obese rats [200] and relieves ectopic lipid accumulation in skeletal muscle and cardiomiocytes [201]. On the other hand, overnutrition, and in particular, high-sucrose diets, enhances BCAAs serum levels via the suppression of protein phosphatase Mg^2+^/Mn^2+^-dependent 1k (PPM1K), favoring IR and cardiometabolic impairments in obese rats [202]. Increased BCAA serum levels may be related to gut microbiome biosynthetic action through *Prevotella copri* and *Bacteroides vulgatus*; *P. copri* or *B. vulgatus* introduction in the mice microbiome can enhance BCAAs production and worsen IR with well-known clinical consequences [203,204]. 

However, it is also important to state that BCAAs are not exclusively harmful: an extensive meta-analysis found a decrease in delayed-onset muscle soreness after exercise in people who underwent BCAAs supplementation versus placebo [205]; moreover, beneficial effects in chronic renal failure have been demonstrated [206].

The use of probiotics or nutritional strategies capable of increasing useful species, while decreasing metabolically unfavorable ones, may be a future path in nutrition.

Finally, reciprocal influences between the gut microbiome and growth hormone have been described: germ-free mice can have a decreased secretion and activity of GH, with reduced IGF-1 levels and impacts on the growth of this animal [207]; the colonization with gut microbial species in such a model and in wild-type rodents induces an increase in IGF-1 concentrations [208]; on the other hand, effects of GH on microbiota have been described [207]. These observations strengthen the role of GH alterations on obesity (see above).

In conclusion, among the pleiotropic activities of adipokines, which justify the term of adipo-brain dialogue, in childhood obesity, the impact on growth and puberty has important clinical consequences. The pathophysiological approach, based on the complex network we have tried to summarize, could be the basis for the personalization of new therapies to counteract the effect of obesity on growth.

How these new theories could be applied to this field is yet to be investigated. As well as IR, leptin resistance and metabolic inflammation can represent targets to work on, with nutritional approaches, to reduce the large problem of childhood obesity.

## Figures and Tables

**Table 1:** Adipokines and their relation to insulin-resistance.

Adipokines	Site of Production	Link with Insulin-Resistance
Leptin	adipose tissue	Leptin expression is stimulated by insulin secretion; Glucose and its metabolites have a triggering role in leptin secretion and expression; Free fatty acids are leptin suppressors.
Resistin	monocyte/macrophages in adipose tissue	Resistin knock-out mice have a reduction in glucose levels by suppression of gluconeogenesis; Chronic “hyperresistinemia” compromises the insulin signaling pathway in all the target tissue: muscle, liver, and adipose tissue [17].
Lipocalin-2	adipose tissue; neutrophils; macrophages; epithelial cells	Lipocalin-2 has a role in the distribution of fat mass, lipid metabolism, and thermoregulation [18,19]; Low levels of lipocalin-2 have shown a protective effect from obesity through the regulation of TNFα and lipoxygenase [20]; High levels of lipocalin-2 are present in MetS [21] but studies in children were conflicting [22,23].
Omentin-1	adipose tissue	Improvement of insulin-mediated glucose uptake in the adipose tissue [24,25,26]; Regulation of food intake [27]; Conflicting results in obesity [24,28,29,30].
Chemerin	liver and adipose tissue	Regulation of adipogenesis; Higher levels both in adult and children obesity; its levels are elevated when visceral adipose tissue is predominant [31,32]; Positive correlation with leptin and negative with adiponectin [33].
Adiponectin	adipose tissue	Insulin-sensitizing activity; Anti-inflammatory and anti-atherogenic; High blood levels are inversely related to obesity, IR, risk of T2DM, dyslipidaemia, and cardiovascular disease; Influence on the expression of key enzymes of gluconeogenesis [34].
Visfatin Other names: *Nicotinamide-phosphorybosiltransferase (NAMPT) or pre-B-cell colony-enhancing factor (PBEF)*	visceral adipose tissue	Serum levels correlate to serum triglycerides levels; Hyper-nutrition grants a down-regulation of its secretion; Stimulation of glucose uptake and suppression of the release of glucose by hepatocytes; Phosphorylation of insulin signaling pathway kinases; In children, it has been associated with BMI, waist circumference, HOMA-IR, and parameters of LGI, with discrepant results [16,35,36,37].
Apelin	stomach, brain, heart, skeletal muscle, and white adipose tissue	It is greatly reduced by fasting. Insulin exerts a positive effect on the direct adipocyte production of apelin [38]; Apelin correlates with states of IR and obesity, stimulates glucose utilization, decreases insulin secretion and catecholamine lipolysis [39].

## Data Availability

Not applicable.

## Abbreviations

ADSF	adipose tissue-specific secretory factor
BOBP	adipocyte-fatty-acid-binding protein
AgRP	agouti-related peptide
AVPV	antero-ventral periventricular nucleus
BCAAs	branched-chain amino acids
BMI	body mass index
C-DII	Children’s Dietary Inflammatory Index
CART	cocaine-and-amphetamine-regulated-transcript
cKD	ketogenic diets
CMKLR1	chemokine-like receptor 1
CNS	central nervous system
CRP	C reactive protein
CSF	cerebro-spinal fluid
DHEA	dehydroepiandrosterone
FXR	farnesoid X receptor
FGF-21	Fibroblast growth factor-21
FIZZ3	found in inflammatory zone 3
GABA	gamma-aminobutyric acid
GH	growth hormone
GnRH	gonadotropin-releasing hormone
HDL	High-density lipoprotein
HL	lateral nucleus
HVM	hypothalamic ventromedial
IGF-1	insulin-like growth factor-1
IGFBP	IGF binding protein
IL	interleukin
IR	insulin resistance
IRS	insulin-related substrate
LCD	low-carbohydrate diet
LCLFD	low-carbohydrate and low-fat diet
LEP-R	leptin receptor
LH	luteinizing hormone
LGI	low-grade inflammation
Mcr	melanocortin receptor
MetS	metabolic syndrome
NAFLD	non-alcoholic fatty liver disease
NAD	nicotinamide adenine dinucleotide
NPY	neuropeptide Y
OS	oxidative stress
POMC	proopiomelanocortin
PPM1K	protein phosphatase Mg^2+^/Mn^2+^-dependent 1k
T2DM	type 2 diabetes mellitus
TCDCA	taurochenodeoxycholic acid
TNF	tumor necrosis factor
TSH	thyroid-stimulating hormone
UCP-1	uncoupling protein-1
WHO	World Health Organization
α-MSH	α-melanocyte-stimulating hormone
